# Do Serum Brain Biomarkers Differentiate the Hemorrhagic Head Injury Lesion Phenotypes? An Interim Analysis of an On-Going Randomized Clinical Trial

**DOI:** 10.3390/biomedicines14030732

**Published:** 2026-03-23

**Authors:** Ayman El-Menyar, Naushad Ahmad Khan, Mohammad Asim, Husham Abdelrahman, Ammar Al-Hassani, Gustav Strandvik, Ashok Parchani, Ahmad Kloub, Sandro Rizoli, Hassan Al-Thani

**Affiliations:** 1 Department of Surgery, Trauma Surgery, Clinical Research, Hamad Medical Corporation, Doha P.O. Box 3050, Qatar; nkhan13@hamad.qa (N.A.K.); masim1@hamad.qa (M.A.); 2 Department of Clinical Medicine, Weill Cornell Medicine, Doha P.O. Box 24144, Qatar; 3 Department of Surgery, Trauma Surgery, Hamad Medical Corporation, Doha P.O. Box 3050, Qatar; hushamco@hotmail.com (H.A.); ammar_alhassani@yahoo.com (A.A.-H.); gstrandvik@hamad.qa (G.S.); aparchani@yahoo.com (A.P.); akloub@hamad.qa (A.K.); srizoli@hamad.qa (S.R.); althanih@hotmail.com (H.A.-T.); 4 Department of Surgery, Qatar University Medical College, Doha P.O. Box 2713, Qatar

**Keywords:** TBI, head injury, brain biomarker, hemorrhagic brain lesion, S-100B, NSE, inflammatory, epinephrine

## Abstract

**Background**: Traumatic head injury (THI) includes a diverse range of hemorrhagic brain lesions (HBL), which are distinct phenotypes with characteristic pathophysiological mechanisms. Computed tomography (CT) is the cornerstone of the initial assessment and diagnosis; however, its sensitivity is limited, especially in mild head injury. Blood-derived biomarkers, including Neuron-Specific Enolase (NSE) and S-100B, have been extensively studied; however, their efficacy in distinguishing HBL subtypes remains unclear. We evaluated whether circulating serum levels of S-100B and NSE can discriminate between distinct intracranial HBLs and extracranial hemorrhagic lesions (ECH). **Methods**: This is an interim analysis of a prospective, randomized, double-blind clinical trial including 434 adult patients with blunt THI. HBL phenotypes identified by CT scan included subarachnoid hemorrhage (SAH), subdural hematoma (SDH), epidural hematoma (EDH), and brain contusion (BC). Unique lesions were considered while overlapping lesions were excluded. Subgaleal hematoma (SGH) was included as an example of ECH. Serum S-100B was assessed within 6 h post-injury, while serum NSE was evaluated at admission, 24 h, and 48 h thereafter. Serum NSE and inflammatory cytokines were quantified in duplicates using a Human Magnetic Luminex 5-plex assay, while serum S-100B concentrations were measured separately. Serum epinephrine concentrations were quantified using an ELISA. Biomarker profiles were analyzed based on lesion phenotype, lesion multiplicity, injury pattern, and clinical outcomes, including hospital length of stay (HLOS) and the Glasgow Outcome Scale—Extended (GOSE). **Results**: Admission median S-100B levels were higher in patients with SAH (495 pg/mL) and lower in those with SGH (191 pg/mL); however, they did not show statistically significant difference among HBL phenotypes. They were significantly higher in patients with polytrauma TBI (420 pg/mL) compared to isolated TBI (258 pg/mL). Baseline and 48 h NSE concentrations were significantly higher in SDH (25,089 and 28,438 pg/mL) than in other THI lesions (*p* = 0.04). There were no statistically significant changes in NSE values over time across all THI lesions except for SDH in which they raised more after 48 h (*p* = 0.02). They had a significant drop in polytrauma over the time (*p* = 0.001). Compared to intracranial lesions, S-100 B levels were significantly lower in SGH and in skull fractures without intracranial hematomas. Both S-100B and NSE levels were elevated in individuals with unfavorable GOSE scores. **Conclusions**: In this secondary exploratory analysis, elevated serum NSE and S-100B levels discriminate between extra- and intracranial lesions and appear to represent distinct but complementary aspects of THI, indicating neuronal damage and its temporal evolution, and predicting clinical and functional outcomes. The present findings reflect association and not causation. Future studies incorporating larger or multicenter cohorts, volumetric imaging, and long-term outcomes are required to validate and refine biomarker-guided algorithms for personalized THI care.

## 1. Introduction

Generally, traumatic head injury (THI) refers to any trauma to the scalp, skull, or brain. Comparatively, traumatic brain injury (TBI) is a specific type of THI where trauma disrupts normal brain function [1]. Although THI and TBI are often used interchangeably, there is a technical distinction in the tissues affected [2]. THI is a major contributor to global mortality and long-term disability. The resultant injuries are heterogeneous in nature and may include hemorrhagic and non-hemorrhagic lesions, both within and outside the brain parenchyma, with micro- and macro-bleeds possible [3,4,5]. Although almost one third of hospitalized head injury patients with normal computed tomography (CT) can have abnormalities on MRI, the use of MRI is limited in acute THI due to complex logistics, potential contraindications, and resource requirements [6,7,8]. If the patient is clinically stable, MRI is mainly reserved for detection of an unresolved diagnosis despite initial CT [9]. While CT is a cornerstone of initial assessment, its diagnostic yield, particularly in mild TBI, can be low because it cannot detect diffuse or microscopic injuries [4,10]. Although 90% of THI admissions are mild TBI, less than 20% of them have demonstrable intracranial injuries on head CT scans [11]. This limitation has spurred significant interest in blood-based biomarkers as adjunctive diagnostic and prognostic tools [12]. There are several benefits from obtaining blood biomarkers in the management pathway of TBI such as better stratification, avoiding unnecessary radiation, justifying advanced imaging, identifying TBI in polytrauma, and supporting further monitoring before discharge from the emergency department [12].

TBI triggers a significant systemic inflammatory response characterized by the sustained release of inflammatory mediators [13]. These molecules play a key role in determining the severity and progression of the injury [14,15]. While biomarkers of TBI are not entirely specific, often reflecting broader physiological processes, the combined measurement of inflammatory and neuronal markers can aid in initial diagnosis, injury severity assessment, and outcome prediction [14,15].

The main inflammatory mediators, including tumor necrosis factor-α (TNF-α), interleukin-6 (IL-6), interleukin-8 (IL-8), and interleukin-10 (IL-10), are thought to influence clinical outcomes following THI [12,16,17]. The immune reaction to TBI is a complex and evolving process, making the detailed study of inflammatory patterns essential. In addition to these markers, the Neuron-Specific Enolase (NSE) and S-100B protein have been extensively investigated [18]. NSE, a cytoplasmic enzyme in neurons, and S-100B, predominantly found in astrocytes, are released into circulation following neuronal and glial injury, respectively [11].

In adults, serum levels of NSE, which has a half-life of approximately 48 h, and S100B, with a half-life of about 2 h, are indicators of neuronal damage and associated with poorer prognosis after TBI [11,19]. However, the timing of these biomarkers’ sampling plays a substantial role in the accuracy of their diagnostic yield [11]. Consequently, diagnostic accuracy may be diminished when sampling is delayed, frequently resulting in false-positive findings. Alternatively, measurements taken at intervals may reflect the temporal progression of pathology or the onset of reparative processes, thereby offering actionable insights for monitoring and individualizing management in vulnerable patient populations [15].

Given the distinct clearance kinetics of NSE and S-100B, as well as the dynamic pathophysiology of TBI, serial biomarker sampling is hypothesized to have superior prognostic utility compared to single measurements. Tracking temporal profiles, such as peak concentration, mean values, and total release, may better characterize the evolving injury cascade and thus more accurately inform clinical staging and outcome prediction [11]. It is noteworthy that S100B, although primarily expressed in the central nervous system, is also found in peripheral nervous tissue, muscle, and fat cells. NSE has been consistently validated across studies as a marker of intracranial injury. S-100B and NSE have been shown to correlate with the extent of damage, including the overall injury burden and CT volumetric assessment of brain lesions [20]. Table 1 summarizes the characteristic features of S-100B and NSE [10,11,19,21,22,23,24,25].

Although elevated levels of both biomarkers correlate with worse outcomes in diffuse TBI, their specific relationship to distinct hemorrhagic brain lesions (HBLs) such as intracerebral and extracranial lesions is poorly understood [14,15]. Examples of intracerebral lesions include subarachnoid hemorrhage (SAH), epidural hematoma (EDH), subdural hematoma (SDH), brain contusion (BC), and diffuse axonal injury (DAI), while extracranial lesions include subgaleal hematoma (SGH) [2,9,26]. These HBLs involve distinct anatomical compartments and pathophysiological mechanisms, which may generate unique biomarker signatures (Table 2) [2,9,26].

Clarifying these patterns could improve diagnostic precision and help differentiate “life-threatening” intracranial/extra axial hematomas from superficial subgaleal hematomas—something the biomarkers can differentiate, and thus, making them useful in clinical practice. Accordingly, an interim analysis of an ongoing prospective clinical trial (BBTBBT) [27] was performed to evaluate whether circulating serum levels of S-100B and NSE can reliably discriminate between distinct HBL types, thereby informing early diagnostic stratification and supporting tailored decision-making. The main study aim was evaluating the role of beta blockers in TBI patients based on the troponin status and their secondary exploratory analyses included the relationship between the biomarkers and head injury lesions [27].

## 2. Materials and Methods

### 2.1. Trial Registration

The primary study (BBTBBT) is registered with ClinicalTrials.gov (Identifier: NCT04508244). This trial was designed and is being conducted in accordance with the CONSORT 2010 guidelines [28] and the approved study protocol [27].

### 2.2. Study Population

Adults with isolated or polytraumatic blunt THI, defined by head Abbreviated Injury Scale (AIS) scores of 1–5 or Glasgow Coma Scale (GCS) scores ranging from 3 to 15, who were enrolled within 24 h of injury, were eligible for inclusion. The present analysis included adult patients across a spectrum of THI severity who underwent computed tomography (CT) at admission to confirm the presence and subtype of HBLs, and who had serum brain biomarker measurements obtained at admission. Detailed inclusion and exclusion criteria are described in previous publications [27]. Briefly, the study included adult patients (≥18 to ≤65 years old) who sustained mild-to-severe blunt TBI (head AIS 1-5 and/or GCS 4-15), screened and enrolled within the first 24 h post-trauma. The study excluded non-survivable injuries (head AIS = 6 and GCS = 3), uncontrolled bleeding, bradycardia, pregnant women, prisoners, and those who required emergency surgery < 6 h and were no longer under the care of the trauma team.

HBL was defined radiologically to reflect a specific hemorrhagic lesion without overlapping with other lesions. Single vs. multiple lesions refers to a single HBL vs. more than one lesion. Isolated head injury means a head injury with head AIS > 2 with or without other regional injury of AIS < 2.

### 2.3. Biomarker Sampling

Serum NSE levels were measured at multiple time points (on-admission NSE-1 (within 6 h), 24 h NSE-2, and 48 h NSE-3). The serum S-100B level was measured at a single time point within 6 h of admission. Additionally, serum levels of pro-inflammatory cytokines (IL-1β, IL-6, IL-8, and IL-18) were measured at three time points: baseline, 24 h, and 48 h after study enrollment. Epinephrine was quantified at baseline and 24 h. Blood samples were obtained in serum separator tubes, clotted at room temperature for 15–30 min, and then centrifuged at 3000 rpm for 15 min. The resulting serum was aliquoted and stored at −80 °C until analysis. Standardized protocols for specimen collection, handling, and processing are recommended by international organizations including the World Health Organization, Clinical and Laboratory Standards Institute, and the Centers for Disease Control and Prevention to minimize hemolysis and improve laboratory quality assurance [29].

### 2.4. Biomarker Assays

Serum NSE and inflammatory cytokines were quantified in duplicates using a Human Magnetic Luminex 5-plex assay (Target: human NSE, IL-1B, IL-6, IL-8, IL-18; Catalogue # LXSAHM-05; R&D Systems, Inc., Minneapolis, MN, USA) and serum S-100B concentrations (Targets: Human S100B; Catalogue # LXSAHM-01; R&D Systems, Inc., Minneapolis, MN, USA) were measured separately as per manufacturer’s instructions. Assay performance characteristics were as follows: For the 5-plex assay, detection ranges and sensitivities were as follows: IL-1β, 17.7–4300 pg/mL (0.8 pg/mL); IL-6, 4.53–1100 pg/mL (1.7 pg/mL); IL-8, 4.12–1000 pg/mL (1.8 pg/mL); IL-18, 7.12–1730 pg/mL (1.93 pg/mL); and NSE, 514–125,000 pg/mL (140 pg/mL). The S-100B 1-plex assay had a detection range of 40.4–9800 pg/mL with a sensitivity of 4.34 pg/mL. Serum epinephrine concentrations were quantified using a commercially available ELISA kit (Catalog No. NBP2-62867; Novus Biologicals Inc., Centennial, CO, USA) with a sensitivity of 18.75 pg/mL and a detection range of 31.25–2000 pg/mL. Experimental data generated from the Human Magnetic Luminex 5-plex and 1-plex assays were processed and analyzed using Bio-Plex Manager software (version 6.2). Absorbance measurements for the serum epinephrine ELISA were acquired using Tecan Magellan™ software (version 1.4.0).

### 2.5. Data Acquisition and Collection

**Figure 1:**
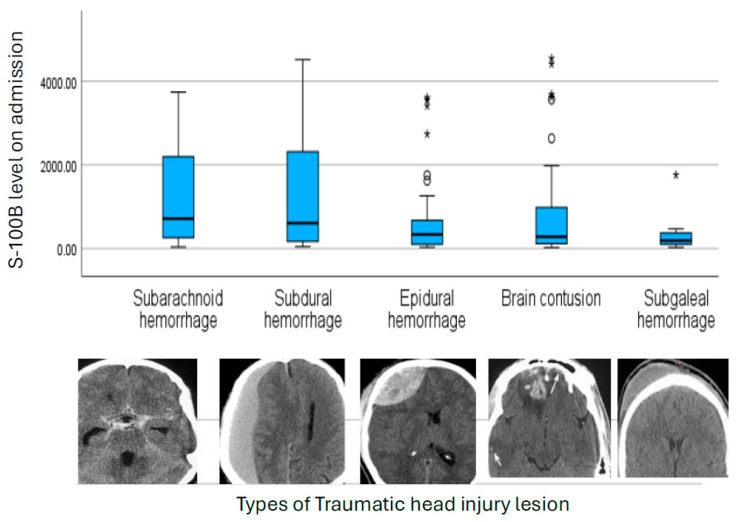
The median on-admission S100B levels based on the intra- and extracranial traumatic head injury lesions.

Data collected included patient demographics, initial vital signs recorded in the trauma resuscitation unit, head AIS scores, ISS, GCS, presence of skull fractures, THI lesions (intracranial and extracranial), hospital length of stay (HLOS), and Glasgow Outcome Scale–Extended (GOSE). Analyses were primarily directed toward evaluating the correlation between serum biomarker levels and HBLs subtype, as well as injury complexity, including single versus multiple lesions, isolated versus polytrauma, and the presence or absence of skull fractures. Definitions of HBLs are detailed in Table 2 and Figure 1 [6,9,26,30,31].

### 2.6. Statistical Analysis

Data for continuous variables are presented as medians with interquartile ranges (IQRs), and categorical variables as counts, and percentages. Descriptive statistics for serum biomarkers are provided for the 5 study groups. The Kolmogorov–Smirnov test assessed whether continuous variables followed a normal distribution or were skewed. Given significant deviations from normality, non-parametric tests were used. Comparison of categorical variables across HBL subtypes and injury groups was performed using the χ^2^ test. Between-group comparisons were conducted using the Mann–Whitney U-test for two-group analyses (e.g., single vs. multiple lesions; isolated vs. polytrauma THI) or the Kruskal–Wallis test for comparisons across more than two lesion types. To evaluate within-group temporal changes in serial biomarkers, the Friedman test for related samples was applied separately within each lesion phenotype and injury-complexity group. When significant overall temporal effects were observed, Wilcoxon signed-rank tests were used for pairwise comparisons between time points, with Bonferroni corrections applied as needed. Primary analyses focused on the association between serum biomarker concentrations and (i) CT-defined HBL phenotypes, (ii) injury complexity (single vs. multiple HBLs), (iii) injury pattern (isolated head injury vs. polytrauma, and (iv) clinical outcomes, including HLOS and GOSE. Given the interim and hypothesis-generating nature of this analysis, no formal multivariable modeling was undertaken. A two-tailed *p*-value < 0.05 was considered statistically significant. All data analysis was conducted using the Statistical Package for the Social Sciences (SPSS), version 21 (IBM Corp., Chicago, IL, USA).

## 3. Results

**Baseline Characteristics and Injury Severity**: This analysis included 434 adult patients with THI, predominantly male (97%), with a mean age of 34.0 ± 4.9 years. Based on the CT scan findings, 149 patients (34.4%) had a single HBL, while 285 patients (65.6%) had multiple HBLs. The unique (not overlapping) hemorrhagic lesions (*n* = 149) included intracranial (SAH (*n* = 21), SDH (*n* = 13), EDH (*n* = 34), BC (*n* = 54)) and extracranial SGH (*n* = 27) lesions. Overall, 188 patients had isolated THI, whereas 246 had polytrauma-associated TBI.

Demographic characteristics were comparable across CT-defined HBL phenotypes, with no significant differences in age, sex and GCS. Neurological status at presentation was relatively comparable across groups and did not significantly differ across hemorrhagic lesion types (*p* = 0.08). In contrast, overall injury burden differed significantly. ISS and head AIS were significantly higher in patients with hemorrhagic lesions, particularly SAH and EDH, compared to SGH, which had the lowest ISS and GCS scores. The proportion of isolated head injury did not differ significantly between lesion types. Patients with multiple intracranial lesions had significantly longer hospital stays compared to those with a single lesion (median 8 vs. 6 days; *p* = 0.003).

**Marker of Sympathetic Stress Response and Inflammatory Cytokines**:

**Epinephrine**: Admission epinephrine levels were uniformly elevated across lesion types; there were no “between-group” differences (*p* = 0.73), indicating a generalized stress response. Epinephrine levels declined significantly at 24 h in SAH (*p* = 0.002) and BC lesions (*p* = 0.001). Epinephrine levels were significantly higher in the isolated compared to the polytrauma lesions on admission; however, their levels did not change significantly over time in both groups.

**Interleukin-6**: Serum IL-6 concentrations were elevated early after injury but did not differ significantly between HBL subtypes at baseline, 24 h, and 48 h. However, within-group analysis showed a significant decline in IL-6 over time in EDH patients (*p* = 0.005), while levels in BC patients remained relatively sustained, suggesting prolonged and sustained parenchymal neuroinflammation. Additionally, IL-6 was significantly higher at baseline in patients with multiple lesions than in those with single lesions (*p* = 0.001), indicating its sensitivity to the overall intracranial injury burden.

**Interleukin-18**: IL-18 levels were consistently elevated across all lesion types without significant differences between HBL subtypes at any time point.

**Interleukin-1β and Interleukin-8**: Both IL-1β and IL-8 showed substantial inter-individual variability but no significant differences across lesion phenotypes, lesion multiplicity, or injury pattern (Table 3).

**Brain Injury Biomarkers**:

**S-100B**: Admission S-100B levels did not differ significantly across HBL subtypes (*p* = 0.09), although patients with SAH showed the highest median concentrations (495 pg/mL), followed by SDH and BC. Levels of S-100B also did not differ between patients with single and multiple brain lesions (*p* = 0.26). In contrast, S-100B was significantly elevated in polytrauma-related TBI compared to isolated head injury (median ~420.4 vs. ~258.6 pg/mL; *p* = 0.002), consistent with extracranial contributions and overall greater systemic injury burden. Patients with less severe trauma, or extracranial head injury such as isolated SGH, exhibited the lowest S-100B levels (*p* = 0.02), as shown in Figure 1.

**Neuron-Specific Enolase (NSE)**: Baseline NSE levels were elevated across all lesion types and differed significantly between HBL phenotypes (*p* = 0.04), with the highest median values in isolated SDH (25,089 pg/mL), lower in SAH (11,576 pg/mL), and the lowest in SGH (9062 pg/mL). NSE levels were higher after 24 h in isolated compared to polytrauma TBI (*p* = 0.03); however, NSE levels did not significantly differ between single and multiple HBLs. NSE levels were higher at admission than at subsequent measurements in polytrauma TBI (*p* = 0.001).

Despite similar baseline levels, the temporal dynamics of NSE diverged by injury severity. Over 48 h, significant NSE declines were observed only in patients with multiple lesions (*p* = 0.05 trend) and polytrauma (*p* = 0.02), whereas levels remained stable in isolated TBI and single-lesion cases (Table 4, Table 5 and Table 6).

**Unfavorable vs. Favorable GOSE**: Functional outcome was measured by GOSE (GOSE ≤ 4 was considered unfavorable). Median serum S-100B levels were higher in patients with unfavorable GOSE (492 [IQR 280-959] vs. 310 [IQR 116-750], *p* = 0.001) compared to favorable GOSE. Serum NSE levels were similar in the first two measurements; however, the third reading was higher in patients with unfavorable GOSE (18,584 [IQR 6755-20483] vs. 11,846 [IQR 6454-24674], *p* = 0.01.

## 4. Discussion

This interim analysis of an ongoing prospective randomized clinical trial assesses the ability of serum NSE and S-100B to differentiate between various traumatic HBL phenotypes, beyond serving merely as generic indicators of brain trauma. The elevated serum S-100B differentiated lesion subtypes but the results were statistically not significant at a single time point. On the other hand, elevated serum NSE levels on admission and after 48 h were significantly able to discriminate these lesions (*p* = 0.04). The behavior of these biomarkers aligns with lesion-specific pathophysiological mechanisms, particularly when lesion burden and overall injury complexity are considered. Temporal biomarker trajectories, particularly of NSE and selected inflammatory mediators, were more informative than absolute concentrations in reflecting injury complexity and secondary injury evolution. The observed 48 h decline in NSE levels could guide the timing of repeat imaging and neurosurgical interventions, ensuring that clinical decisions align with the typical windows for such procedures. Furthermore, injury burden, reflected as multiple HBLs or polytrauma, had a greater influence on biomarker dynamics than the CT-defined lesion phenotype alone.

Overall, S-100B reflected global injury burden rather than specific hemorrhagic lesion phenotype. In our cohort, S-100B demonstrated a graded increase across HBL phenotypes, with the highest baseline concentrations observed in SAH, and the lowest in SGH. This pattern is mechanistically plausible and consistent with the known biology of S-100B [21,32,33,34]. SAH represents a diffuse cortical insult characterized by widespread astroglial activation and changes in BBB permeability, both of which facilitate rapid S-100B efflux into the systemic circulation [35,36]. In contrast, SGH is extracranial and lacks direct parenchymal or BBB involvement, explaining the comparatively lower S-100B levels [37]. Although overlap between lesion groups precluded statistical discrimination, the directional differences observed support the concept that baseline S-100B preferentially reflects the extent of astroglial activation and BBB disruption rather than focal hemorrhage volume or lesion compartment. Table 7 summarizes the serum S-100B and NSE profiles in this interim analysis.

Serum S-100B exhibits rapid kinetics with a very short biological half-life (30–120 min), rising quickly following head injury and declining unless ongoing tissue damage is present [3,38]. Consequently, its prognostic value is greatest in the early post-injury period. Prior studies indicate that S-100B is most informative within the first 12–24 h after TBI [3,20]. In this context, the single admission measurement in our study was well-suited to capture the acute peak of S-100B release. However, the lack of serial measurements precluded assessment of subsequent declines or secondary elevations, which could reflect secondary astroglial injury or delayed BBB dysfunction, which may be particularly relevant in lesions such as contusions or evolving SDH [39].

In contrast to S-100B, the NSE peaked more gradually, often remaining high or even going up during the first 24 h, reflecting secondary neuronal injury kinetics [34,40]. Baseline NSE levels were elevated across all HBL phenotypes, without a significant ‘between-group’ difference, suggesting that acute neuronal injury is a common denominator across HBL subtypes, irrespective of whether the bleeding is extra-axial or intra-parenchymal.

By comparison, early NSE increases were most significant in SDHs, indicating considerable neuronal damage. Notably, even at later time points, lesion-specific tendencies were observed, with NSE levels remaining elevated in SDH, SAH, and BCs, suggesting a prolonged neuronal injury process in these lesion types. Although not statistically significant, this pattern is consistent with the hypothesis that neuronal injury in hemorrhagic TBI is driven more by mass effect, cortical strain, and secondary pathophysiology rather than hemorrhage location alone [20].

Thus, the persistence or delayed normalization of NSE in these lesions may serve as a surrogate for ongoing neuronal stress not readily captured by static CT imaging [34,40]. In contrast, parenchymal contusions represent direct neuronal disruption, which may explain their relatively high baseline. Nevertheless, the later NSE peaks in SDH and SAH warrant further study of secondary injury cascades.

Interestingly, NSE demonstrated lesion-burden-dependent temporal dynamics, with a significant decline over 48 h in patients with multiple HBLs and a more modest, non-significant reduction in single-lesion TBI patients. This suggests greater initial neuronal injury with more extensive hemorrhage, followed by gradual resolution, whereas single lesions may induce smaller but more persistent neuronal insults. Nonetheless, NSE levels converged across all groups by 48 h, suggesting resolution of acute injury and the initiation of clearance mechanisms and secondary injury processes, consistent with previous studies’ findings [40,41].

In our cohort, injury patterns significantly affected the biomarker profile. Polytrauma patients had substantially higher S-100B levels than those with isolated head injuries, suggesting its marked sensitivity to systemic trauma. This observation aligns with a recent analysis from CENTER-TBI, which showed that S100B correlates more strongly with global injury burden and extracranial trauma than with specific intracranial lesion morphology [20].

This heightened sensitivity can be a double-edged sword. On the one hand, it enhances the clinical utility of S-100B as an early screening biomarker for intracranial injury, especially given its high negative predictive value in mild TBI. However, it also diminishes specificity due to possible extracranial sources (muscle, bone, and adipose tissue), as indicated by its positive correlation with overall injury severity [11,21,41].

In contrast, NSE appeared relatively more CNS-specific in the polytrauma context, with comparable acute levels observed in isolated and polytraumatic head injury. This differential response in polytrauma (elevated S-100B but not NSE) underscores the need for biomarker panels and cautious S-100B interpretation in multi-injured patients. Despite these caveats, both biomarkers exhibited significant predictive utility.

S-100B has consistently demonstrated superior sensitivity and specificity compared to NSE for identifying CT-visible intracranial damage, making it more beneficial for early diagnostic and prognostic assessment in THI [11]. The rapid release kinetics support current guidelines for sampling within six hours post-injury [38], whereas the prolonged half-life of NSE facilitates its application for late-phase evaluation [11,34,41]. A serum S-100B concentration below 0.10 µg/L in this initial phase possesses an exceptionally high negative predictive value for excluding clinically significant CT lesions, thereby facilitating safe triage in mild head injury [10,42].

Epinephrine levels were uniformly elevated at admission across lesion types, reflecting a generalized sympathetic stress response [14,43,44]. Serial measurements showed that epinephrine levels decreased after 24 h in single-lesion patients. In contrast, they remained elevated in multiple-lesion and polytrauma cases, suggesting prolonged neuroendocrine activation with increasing injury complexity [42,43,44]. Although epinephrine is not brain-specific, its temporal profile provides complementary insight into systemic stress and the risk of secondary injury [43,44].

The inflammatory response following THI is a major catalyst of secondary damage [14]. We found a correlation between IL-6 and IL-18 and injury complexity, which is consistent with prior studies on THI patients [14,45,46,47]. IL-6 was elevated early across all lesion types but varied after 48 h, remaining consistently higher in BC patients. This probably indicates persistent parenchymal inflammation and microvascular damage in contusions [45,46,47], unlike extra-axial lesions such as EDH, SDH, and SAH, in which IL-6 decreased markedly over time. Previous studies have shown similar patterns linking IL-6 persistence to the severity of parenchymal damage [19,22,45,46,47]. Moreover, IL-18 levels were elevated across all groups but remained considerably higher after 48 h in individuals with multiple HBLs, indicating a correlation with cumulative cerebral injury rather than lesion phenotype. An elevated IL-18 level reflects inflammasome activation and neuro-immune signaling after severe TBI [14,45].

From a clinical perspective, our study reinforces the potential value of both biomarkers while clarifying their limitations. Despite comparable admission GCS scores, patients with SDH, BC, multiple HBLs, and polytrauma had significantly longer HLOS and a trend toward worse functional outcomes, suggesting that these two biomarkers quantitatively mirror injury severity and can prognosticate recovery to some extent. Patients with very high S-100B on admission or with sustained NSE elevations were more likely to have severe brain injury, longer ICU/hospital stays, and worse functional status at discharge. Such information could be useful for early risk stratification. Specifically, both NSE and S-100B were higher in patients with unfavorable outcomes, reinforcing their prognostic relevance [20,21,34,45,47].

**Limitations**: Several limitations warrant careful consideration. Most patients included in this interim analysis had mild TBI, which may have attenuated biomarker amplitude and temporal variability, thereby limiting the ability to detect robust associations with specific HBL phenotypes. Consequently, the observed biomarker trajectories may not fully capture the dynamic evolution of injury in more severe TBI. Additional limitations include small sample sizes in some lesion subgroups, particularly SAH and SDH, which may have reduced statistical power for lesion-specific comparisons. We attempted to isolate distinct HBLs without overlapping with other lesions, which explains the relatively small sample size but ensures reliable results to some extent. Notably, this interim analysis has been made on only 57% of the targeted study population (*n* = 434/772). 

In addition, a CT-based lesion does not capture lesion volume, diffuse axonal injury, or micro-bleeds, which may influence biomarker release. Therefore, lesions were anatomically defined but not stratified by radiological severity (e.g., volume, mass effect, midline shift) or by surgical management. DAI was not included in the analysis as few individual cases were documented (*n* = 7). Furthermore, S-100B measurements were taken only at admission. This could be due to a financial constraint and to the marker’s short half-life. Integrating these biomarkers with CT scan imaging would be of value to better triaging and risk stratification of patients with THI. Cost-effectiveness analysis is warranted in this regard as well.

The current study relied on two brain biomarkers; however, newer markers such as Glial Fibrillary Acidic Protein (GFAP) and the neuronal Ubiquitin C-terminal hydrolase L1 (UCH-L1) were not included. Of note, the outcomes of studies on these novel biomarkers remain debatable [5,48,49]. One study showed no significant differences in GFAP and UCH-L1 concentrations between patients with isolated head injury and those with non-isolated head injury while another study showed that day-of-injury levels of GFAP and UCH-L1 have a prognostic value for predicting unfavorable outcomes, but not in predicting incomplete recovery at 6 months [48,49]. Moreover, no studies have reported a correlation between the type of lesion and these novel markers.

As there is no multivariable analysis conducted with confounders adjustment, the results should be considered cautiously, as they reflect an association rather than causal relationship. This study does not report any correction for multiple testing despite conducting several statistical comparisons, which increases the risk of type I error. However, the present analysis highlighted the need for conducting multicenter studies to validate these findings and establish a clearer understanding of the behavior of brain biomarkers across different types of hemorrhagic head lesion. This will provide better triage and management pathways for patients with THI.

## 5. Conclusions

These secondary exploratory findings demonstrate that serum NSE and S-100B show distinct patterns across HBL phenotypes, suggesting that they can provide more than a generic injury signal and may function as dynamic indicators of the severity and prognosis of traumatic hemorrhagic brain injury. S-100B is particularly sensitive to astroglial injury and systemic trauma, whereas NSE provides insight into neuronal injury kinetics and secondary damage. These biomarkers can discriminate extra- and intracranial THI lesions and flag the presence of intracranial bleeding when a skull fracture or subgaleal lesion is present. However, these findings are preliminary and support integrating serial, multimodal biomarker profiling with imaging and clinical assessment to improve diagnostic precision and prognostication in THI. Notably, the present findings reflect association rather than causation. Future studies incorporating larger or multicenter cohorts, volumetric imaging, and long-term outcomes are required to validate and refine biomarker-guided algorithms for personalized THI care.

## Figures and Tables

**Table 1:** Comparative characteristics of serum brain injury biomarkers S-100B and Neuron-Specific Enolase (NSE).

Feature	S-100B	Neuron-Specific Enolase (NSE)
**Biological origin**	Predominantly astrocytes; released actively or passively following astroglial injury	Neurons; released following neuronal injury and blood–brain barrier (BBB) disruption
**Serum half-life**	Short (30 min to 2 h)	Longer (up to ≈48 h)
**Mechanism of release**	Astroglial damage and BBB disruption	Neuronal cell injury with BBB compromise
**Extracranial sources/specificity limitations**	Specificity limited by extracranial expression (Langerhans cells, adipocytes, epithelial cells, cardiac and skeletal muscle, chondrocytes)	Specificity limited by hemolysis; erythrocytes contain high NSE concentrations, leading to false elevation in polytrauma
**Clinical significance**	Reflects extent of astroglial injury; strong early predictor of mortality in isolated TBI	Potent prognostic marker. Persistent or secondary elevation reflects ongoing neuronal damage and secondary injury
**Diagnostic thresholds**	>0.13 µg/L suggests head injury; >0.50 µg/L associated with moderate to severe TBI	No universally accepted cut-off. Interpretation depends on timing and hemolysis control
**Relation to BBB integrity**	CSF concentrations may be up to 100-fold higher than serum; highly sensitive indicator of BBB disruption	Serum release closely linked to BBB disruption; CSF-to-serum gradient less pronounced
**Pathophysiological role**	Concentration-dependent effects: neuroprotective at low levels, pro-inflammatory and neurotoxic at high levels	Marker of structural neuronal damage rather than mediator
**Kinetic considerations**	Rapid rise and clearance favor early (<6 h) sampling	Longer half-life allows later assessment but may delay washout of extracranial contributions
**Diagnostic performance (meta-analysis)**	Sensitivity 80%, specificity 59% for CT-detected intracranial injury; sensitivity 83%, specificity 51% for unfavorable outcome	Sensitivity 74%, specificity 46% for CT-detected injury (AUC ~0.66); sensitivity 80%, specificity 59% for unfavorable outcome
**Systemic clearance**	Renally eliminated; baseline levels higher in renal failure (mild–moderate renal dysfunction typically does not significantly affect levels)	Extracranial contribution from trauma-related hematomas possible; clearance influenced by hemolysis and injury burden
**Overall utility in TBI**	Superior early diagnostic and rule-out biomarker; high negative predictive value	Valuable for prognostication and longitudinal monitoring of neuronal injury

**TBI:** traumatic brain injury.

**Table 2:** Definitions and radiological characteristics of HBL phenotypes in traumatic head injury.

Lesion Type	Anatomical Location	Primary Mechanism	Characteristic CT Findings	Key Clinical/Pathophysiological Features
**SAH**	Extra-axial; blood within the subarachnoid space, tracking along sulci and basal cisterns	Tearing of cortical arteries and veins; rupture of cortical contusions	Hyperdense blood following sulcal and cisternal contours	Most common extra-axial hemorrhage; reflects diffuse cortical injury, meningeal irritation, and blood–brain barrier disruption; often associated with secondary vasospasm and widespread astroglial activation
**EDH**	Extra-axial; between skull and dura mater	Typically, arterial injury (~90%), commonly middle meningeal artery	Biconvex (lentiform), hyperdense collection; does not cross suture lines	Frequently associated with skull fractures (~90%), especially temporal bone fractures; may exhibit rapid expansion and mass effect
**SDH**	Extra-axial; between dura and arachnoid mater	Tearing of bridging cortical veins	Crescent-shaped hyperdense collection; may cross suture lines but not dural attachments	Commonly associated with acceleration–deceleration injuries; may evolve subacutely or chronically; significant contributor to secondary neuronal injury
**BC**	Intra-axial; brain parenchyma (commonly orbitofrontal and temporal lobes)	Sudden deceleration with forceful impact of brain against osseous surfaces	Multiple focal hyperdense areas with surrounding edema	Most common intra-axial injury; represents direct neuronal and microvascular disruption; strongly associated with neuroinflammation and secondary injury cascades
**SGH**	Extracranial; potential space between galea aponeurotica and pericranium	Traumatic shearing separating aponeurosis from periosteum	Diffuse scalp swelling; crosses cranial suture lines	Not a primary brain parenchymal injury; lacks BBB involvement; may coexist with skull fractures; lowest expected CNS biomarker release

**HBL:** hemorrhagic brain lesion; **SAH:** subarachnoid hemorrhage; **EDH:** epidural hematoma; **SDH:** subdural hematoma; **BC:** brain contusion; **SGH:** subgaleal hematoma.

**Table 3:** Comparison of inflammatory markers based on CT-based head injury lesions (*n* = 149).

Variables	Subarachnoid Hemorrhage (SAH) *n* = 21	Subdural Hematoma (SDH) *n* = 13	Epidural Hematoma (EDH) *n* = 34	Brain Contusion (BC) *n* = 54	Subgaleal Hematoma (SGH) *n* = 27	*p*-Value
**Age years**	39.6 ± 10.9	36.8 ± 10.2	32.5 ± 8.4	36.0 ± 10.5	34.6 ± 9.6	0.14
**Males**	21 (100%)	13 (100%)	32 (94.1%)	53 (98.1%)	27 (100%)	0.42
**Injury severity score**	19.3 ± 9.9	17.2 ± 5.6	22.1 ± 10.7	18.6 ± 9.7	12.9 ± 6.8	0.005
**Glasgow coma score**	14 (6.5–15.0)	14 (8.5–15.0)	15 (11.5–15.0)	13.5 (6.0–15.0)	15 (11.0–15.0)	0.08
**Head AIS**	3.5 ± 1.0	3.3 ± 0.7	3.4 ± 1.1	3.0 ± 0.9	2.4 ± 1.1	0.001
**Isolated head injury**	10 (47.6%)	5 (41.7%)	12 (35.3%)	23 (42.6%)	10 (37.0%)	0.90
**Interleukin-6 (pg/mL)**						
Baseline (on admission)	28.1 (12.5–52.1)	49.4 (8.3–80.1)	37.3 (19.8–87.9)	31.4 (19.1–59.2)	19.1 (14.2–45.1)	0.42
After 24 h	20.0 (12.2–34.4)	27.9 (12.9–87.5)	27.5 (10.5–72.9)	33.68 (15.5–76.6)	23.8 (13.2–52.9)	0.53
After 48 h	13.9 (8.1–44.8)	11.2 (6.2–45.9)	21.4 (10.6–39.6)	34.2 (12.7–77.3)	27.7 (15.2–58.1)	0.12
*p* value within the group	0.41	0.55	0.005	0.42	0.84	
**Interleukin-18 (pg/mL)**						
Baseline (on admission)	280.7 (223.9–632.1)	284.5 (194.3–421.7)	259.9 (194.8–797.7)	334.4 (191.2–800.2)	555.1 (236.9–770.9)	0.62
After 24 h	318.2 (211.1–553.9)	343.3 (222.9–528.5)	253.0 (196.0–667.3)	307.4 (205.6–753.8)	653.4 (257.4–906.2)	0.33
After 48 h	395.8 (216.2–811.7)	347.1 (212.0–783.5)	236.5 (172.3–773.9)	357.5 (161.6–914.4)	376.3 (237.1–776.3)	0.81
*p* value within the group	0.62	0.33	0.23	0.72	0.08	
**Interleukin-1β (pg/mL)**						
Baseline (on admission)	15.3 (5.4–22.7)	17.9 (12.0–32.1)	17.0 (10.5–53.4)	17.0 (14.3–24.3)	18.4 (16.6–40.6)	0.34
After 24 h	7.1 (4.7–20.4)	16.4 (10.7–47.8)	16.5 (6.6–49.8)	16.9 (7.7–43.1)	13.6 (8.8–40.4)	0.19
After 48 h	6.6 (5.4–16.5)	15.4 (7.9–24.8)	14.9 (9.3–23.5)	13.9 (7.1–18.9)	15.9 (6.4–43.1)	0.19
*p* value within the group	0.23	0.33	0.30	0.03	0.27	
**Interleukin-8 (pg/mL)**						
Baseline (on admission)	85.3 (22.6–308.6)	53.8 (18.5–284.7)	64.9 (22.1–279.1)	88.1 (43.0–295.8)	77.1 (19.8–960.0)	0.60
After 24 h	55.1 (16.9–664.8)	41.5 (29.5–1102.7)	83.9 (24.8–153.8)	71.7 (36.8–160.1)	158.6 (39.0–887.9)	0.52
After 48 h	70.2 (26.8–623.5)	60.5 (26.5–119.7)	85.5 (32.7–393.3)	116.2 (27.5–635.1)	95.4 (35.2–813.3)	0.74
*p* value within the group	0.29	0.77	0.36	0.49	0.55	

**Table 4:** Comparison of brain injury markers based on CT-based head injury lesions (*n* = 149).

Variables	Subarachnoid Hemorrhage *n* = 21	Subdural Hematoma *n* = 13	Epidural Hematoma *n* = 34	Brain Contusion *n* = 54	Subgaleal Hematoma *n* = 27	*p*-Value
**Brain injury markers**						
**S-100B (pg/mL)** at baseline	495 (216–2125)	357 (140–1897)	332 (84–791)	282 (117–1001)	191 (88–381)	0.09
**Neuron-Specific Enolase (pg/mL) (514–125,000)**						
Baseline (on admission)	11,576 (8313–17,086)	25,089 (13,703–41,835)	18,202 (8356–36,873)	13,964 (7406–32,208)	9062 (4877–20,935)	0.04
After 24 h	12,951 (6347–17,341)	21,519 (11,023–35,757)	13,071 (6292–10,196)	11,838 (7684–22,789)	10,425 (5307–22,214)	0.43
After 48 h	15,205 (7054–20,763)	28,438 (22,433–55,582)	11,407 (5988–28,035)	15,404 (8781–22,391)	8668 (5785–21,368)	0.04
*p* value within the group *	1.00	0.02	0.41	0.49	0.28	
**Epinephrine (pg/mL)**						
Baseline (on admission)	1028 (274–2210)	1505 (326–2528)	487 (181–1980)	578 (266–2795)	1468 (342–2448)	0.73
After 24 h	612 (227–1610)	847 (335–2474)	1043 (222–2436)	540 (210–1540)	1355 (429–2134)	0.16
*p* value within the group *	0.002	0.78	0.17	0.001	0.31	

NSE reference range: ≤16.3 µg/L (≤16,300 pg/mL); continuous variables presented as median and IQR; * Wilcoxon signed-rank tests for K-related samples (*p* value determined by Friedman) was used to see significant differences from baseline to 48 h within the group; Kruskal–Wallis test was used to see significant differences between more than two groups.

**Table 5:** Comparison of brain injury and inflammatory markers based on single versus multiple brain injury lesions (*n* = 434).

Variables	Single Lesion (*n* = 149)	Multiple Lesions (*n* = 285)	*p* Value **
**Brain injury markers**			
**S-100B (pg/mL)** at baseline	326 (117–796)	378 (133–997)	0.26
**Neuron-Specific Enolase (pg/mL)**			
Baseline (on admission)	14,407 (7395–28,114)	18,185 (7847–35,041)	0.15
After 24 h	12,689 (6807–23,447)	13,557 (8368–27,468)	0.21
After 48 h	14,806 (7445–26,453)	15,792 (7402–34,049)	0.51
*p* value within the group *	0.54	0.05	
**Pro-inflammatory cytokines**			
**Interleukin-6 (pg/mL)**			
Baseline (on admission)	30.7 (16.3–60.8)	45.1 (22.6–97.3)	0.001
After 24 h	27.2 (13.6–60.9)	32.6 (15.9–71.1)	0.10
After 48 h	22.9 (10.4–51.5)	24.4 (9.6–57.0)	0.57
*p* value within the group *	0.01	0.001	
**Interleukin-18 (pg/mL)**			
Baseline (on admission)	292 (201–739)	371 (197–776)	0.32
After 24 h	322 (207–734)	400 (199–764)	0.45
After 48 h	346 (189–814)	462 (209–847)	0.18
*p* value within the group *	0.59	0.01	
**Interleukin-1β (pg/mL)**			
Baseline (on admission)	17.5(12.6–30.6)	17.6 (10.8–33.0)	0.93
After 24 h	14.8 (6.9–43.2)	15.7 (6.5–35.7)	0.88
After 48 h	13.5 (6.4–20.4)	14.6 (6.8–27.9)	0.44
*p* value within the group *	0.001	0.001	
**Epinephrine (pg/mL)**			
Baseline (on admission)	729 (285–2365)	919 (322–2151)	0.61
After 24 h	772 (259–1847)	869 (332–2454)	0.45
*p* value within the group *	0.001	0.31	
**GOSE**	8 (8–8)	8 (7–8)	0.24
**Hospital LOS**	6 (3–12.5)	8 (5–21)	0.003

Data presented as median and IQR; * Friedman Test was used for K-related samples to see significant differences from baseline to 48 h within the group (if needed Wilcoxon signed-rank tests with Bonferroni correction); ** Mann–Whitney U tests were used to see significant differences between the two groups.

**Table 6:** Comparison of brain injury and inflammatory markers based on isolated versus polytrauma brain injury (*n* = 434).

	Isolated Head Injury (*n* = 188)	Polytrauma TBI (*n* = 246)	*p* Value
**S-100B (pg/mL)** at baseline	258.6 (116.8–688.1)	420.4 (141.8–1531.8)	0.002
**Neuron-Specific Enolase (pg/mL)**			
Baseline (on admission)	15,726 (7493–33,367)	17,593 (7660–32,252)	0.88
After 24 h	15,366 (7128–34,259)	12,091 (7264–22,355)	0.03
After 48 h	15,698 (7372–31,394)	12,890 (6696–24,584)	0.09
*p* value within the group	0.73	0.001	
**Epinephrine (pg/mL)**			
Baseline (on admission)	1064 (373–1064)	679 (267–2062)	0.038
After 24 h	989 (409–2454)	736 (265–2032)	0.095
*p* value within the group	0.21	0.14	

**Table 7:** Summary of serum S-100B and NSE profiles in patients with traumatic head injury.

Comparison Domain	S-100B (Trend Profile)	NSE (Trend Profile)	Interpretation
**HBL phenotype**	Highest early levels in SAH; lower in focal lesions; lowest in SGH; no significant between-phenotype discrimination	Early elevations most pronounced in BC and SDH; persistent elevation in SDH/EDH at later time points	S-100B reflects diffuse astroglial/BBB injury; NSE reflects focal neuronal damage
**Temporal pattern**	Rapid early peak; Kinetic trend not measured in the study.	Gradual peak with prolonged elevation (up to 48 h) followed by decline	Distinct kinetics: S-100B = early signal; NSE = delayed neuronal injury
**Isolated head injury**	Lower early levels	Similar baseline NSE compared with polytrauma	S-100B sensitive to extracranial injury; NSE more CNS-specific
**Polytrauma TBI**	Significantly higher early levels	No significant acute increase vs. isolated TBI	Extracranial contribution affects S-100B interpretation
**Single HBL**	Comparable early levels	Modest decline over time	Less cumulative neuronal injury
**Multiple HBLs**	Similar early S-100B levels	Higher initial NSE with significant decline over 48 h	NSE reflects cumulative neuronal burden
**Clinical severity/outcome**	Higher in unfavorable GOSE	Higher and more persistent elevations	Both biomarkers prognostic, at different phases

## Data Availability

Data will be available on reasonable request from the PI after approval of the medical research center, Doha, Qatar.

## Abbreviations

THI	Traumatic head injury
TBI	Traumatic brain injury
HBL	Hemorrhagic brain lesion
NSE	Neuron-Specific Enolase
S-100B	Calcium-binding protein
CT scan	Computerized tomography
IL	Interleukins
GCS	Glasgow Coma Scale
AIS	Abbreviated Injury Scale
GOSE	Glasgow Outcome Scale–Extended

## References

[1] Silver J.M., McAllister T.W., Yodofsky S.C. (2005). Textbook of Traumatic Brain Injury.

[2] Tse K.M., Lim S.P., Tan V.B.C., Lee H.P. (2014). A Review of Head Injury and Finite Element Head Models. Am. J. Engineering. Technol. Soc..

[3] Thelin E.P., Nelson D.W., Bellander B.M. (2017). A review of the clinical utility of serum S100B protein levels in the assessment of traumatic brain injury. Acta Neurochir..

[4] Irimia A., Van Horn J.D., Vespa P.M. (2018). Cerebral microhemorrhages due to traumatic brain injury and their effects on the aging human brain. Neurobiol. Aging.

[5] Haley K.E., Greenberg S.M., Gurol M.E. (2013). Cerebral microbleeds and macrobleeds: Should they influence our recommendations for antithrombotic therapies?. Curr. Cardiol. Rep..

[6] Wintermark M., Sanelli P.C., Anzai Y., Tsiouris A.J., Whitlow C.T. (2015). Imaging evidence and recommendations for traumatic brain injury: Conventional neuroimaging techniques. J. Am. Coll. Radiol..

[7] Steyerberg E.W., Wiegers E., Sewalt C., Buki A., Citerio G., de Keyser V., Ercole A., Kunzmann K., Lanyon L., Lecky F. (2019). Case-mix, care pathways, and outcomes in patients with traumatic brain injury in CENTER-TBI: A European prospective, multicentre, longitudinal, cohort study. Lancet Neurol..

[8] Lee H., Yang Y., Xu J., Ware J.B., Liu B. (2021). Use of Magnetic Resonance Imaging in Acute Traumatic Brain Injury Patients is Associated with Lower Inpatient Mortality. J. Clin. Imaging Sci..

[9] Currie S., Saleem N., Straiton J.A., Macmullen-Price J., Warren D.J., Craven I.J. (2016). Imaging assessment of traumatic brain injury. Postgrad. Med. J..

[10] Zhang J., Puvenna V., Janigro D., Laskowitz D., Grant G. (2016). Frontiers in Neuroscience Biomarkers of Traumatic Brain Injury and Their Relationship to Pathology. Translational Research in Traumatic Brain Injury.

[11] Zarei H., Roshdi Dizaji S., Toloui A., Yousefifard M., Esmaeili A. (2024). Diagnostic and Prognostic Values of S100B versus Neuron Specific Enolase for Traumatic Brain Injury; a Systematic Review and Meta-analysis. Arch. Acad. Emerg. Med..

[12] Hossain I., Marklund N., Czeiter E., Hutchinson P., Buki A. (2023). Blood biomarkers for traumatic brain injury: A narrative review of current evidence. Brain Spine.

[13] Li L.M., Kodosaki E., Xu C.J.Y., Heslegrave A., Zetterberg H., Graham N.S.N., Zimmerman K.A., Garbero E., Moro F., Magnoni S. (2026). Systemic inflammation and its associations in acute moderate-severe Traumatic Brain Injury: A cross-sectional study. Brain Behav. Immun. Health.

[14] El-Menyar A., Asim M., Khan N., Rizoli S., Mahmood I., Al-Ani M., Kanbar A., Alaieb A., Hakim S., Younis B. (2024). Systemic and cerebro-cardiac biomarkers following traumatic brain injury: An interim analysis of randomized controlled clinical trial of early administration of beta blockers. Sci. Rep..

[15] Pryzmont M., Kosciuczuk U., Maciejczyk M. (2025). Biomarkers of traumatic brain injury: Narrative review and future prospects in neurointensive care. Front. Med..

[16] McKee C.A., Lukens J.R. (2016). Emerging Roles for the Immune System in Traumatic Brain Injury. Front. Immunol..

[17] Yousefzadeh-Chabok S., Dehnadi Moghaddam A., Kazemnejad-Leili E., Saneei Z., Hosseinpour M., Kouchakinejad-Eramsadati L., Razzaghi A., Mohtasham-Amiri Z. (2015). The Relationship Between Serum Levels of Interleukins 6, 8, 10 and Clinical Outcome in Patients With Severe Traumatic Brain Injury. Arch. Trauma. Res..

[18] Park S.H., Hwang S.K. (2018). Prognostic Value of Serum Levels of S100 Calcium-Binding Protein B, Neuron-Specific Enolase, and Interleukin-6 in Pediatric Patients with Traumatic Brain Injury. World Neurosurg..

[19] Cheng F., Yuan Q., Yang J., Wang W., Liu H. (2014). The prognostic value of serum neuron-specific enolase in traumatic brain injury: Systematic review and meta-analysis. PLoS ONE.

[20] Whitehouse D.P., Monteiro M., Czeiter E., Vyvere T.V., Valerio F., Ye Z., Amrein K., Kamnitsas K., Xu H., Yang Z. (2022). Relationship of admission blood proteomic biomarkers levels to lesion type and lesion burden in traumatic brain injury: A CENTER-TBI study. eBioMedicine.

[21] Koivikko P., Katila A.J., Takala R.S., Hossain I., Luoto T.M., Raj R., Koivisto M., Tenovuo O., Blennow K., Hutchinson P. (2025). Blood biomarkers to identify patients with different intracranial lesion combinations after traumatic brain injury. Brain Spine.

[22] Langeh U., Singh S. (2021). Targeting S100B Protein as a Surrogate Biomarker and its Role in Various Neurological Disorders. Curr. Neuropharmacol..

[23] Martin R.M., Wright M.J., Lutkenhoff E.S., Ellingson B.M., Van Horn J.D., Tubi M., Alger J.R., McArthur D.L., Vespa P.M. (2017). Traumatic hemorrhagic brain injury: Impact of location and resorption on cognitive outcome. J. Neurosurg..

[24] Stefanović B., Đurić O., Stanković S., Mijatović S., Doklestić K., Stefanović B., Jovanović B., Marjanović N., Kalezić N. (2017). Elevated Serum Protein S100B and Neuron Specific Enolase Values as Predictors of Early Neurological Outcome After Traumatic Brain Injury. J. Med. Biochem..

[25] Sundararajan T., Tesar G.E., Jimenez X.F. (2016). Biomarkers in the diagnosis and study of psychogenic nonepileptic seizures: A systematic review. Seizure.

[26] Chen C.E., Liao Z.Z., Lee Y.H., Liu C.C., Tang C.K., Chen Y.R. (2017). Subgaleal Hematoma at the Contralateral Side of Scalp Trauma in an Adult. J. Emerg. Med..

[27] El-Menyar A., Asim M., Bahey A.A., Chughtai T., Alyafai A., Abdelrahman H., Rizoli S., Peralta R., Al-Thani H. (2021). Beta blocker use in traumatic brain injury based on the high-sensitive troponin status (BBTBBT): Methodology and protocol implementation of a double-blind randomized controlled clinical trial. Trials.

[28] Schulz K.F., Altman D.G., Moher D. (2010). CONSORT 2010 statement: Updated guidelines for reporting parallel group randomised trials. BMJ (Clin. Res. Ed.).

[29] CDC (2024). Centers for Disease Control Prevention Laboratory Quality Assurance Standardization Programs.

[30] Tenny S., Das J.M., Thorell W. (2024). Intracranial Hemorrhage Overview. StatPearls [Internet].

[31] Pagkou D., Papavramidis T., Mavropoulou X., Moysidis M., Patsalas I. (2021). Massive subgaleal hematoma in a 62-year-old man treated with apixaban as a consequence of mild head trauma. Folia Medica.

[32] Laribi S., Kansao J., Borderie D., Collet C., Deschamps P., Ababsa R., Mouniam L., Got L., Leon A., Thoannes H. (2014). S100B blood level measurement to exclude cerebral lesions after minor head injury: The multicenter STIC-S100 French study. Clin. Chem. Lab. Med..

[33] Poislane P.A., Papin M., Masson D., Goffinet N., David A., Le Bastard Q., De Carvalho H. (2025). Diagnostic performance of S100B assay for intracranial hemorrhage detection in patients with mild traumatic brain injury under antiplatelet or anticoagulant therapy. Sci. Rep..

[34] Thelin E.P., Jeppsson E., Frostell A., Svensson M., Mondello S., Bellander B.M., Nelson D.W. (2016). Utility of neuron-specific enolase in traumatic brain injury; relations to S100B levels, outcome, and extracranial injury severity. Crit. Care.

[35] Yu K., Wang D., Yu W. (2025). Astrocyte-microglia crosstalk in subarachnoid hemorrhage: Mechanisms and treatments. Front. Immunol..

[36] Solár P., Zamani A., Lakatosová K., Joukal M. (2022). The blood-brain barrier and the neurovascular unit in subarachnoid hemorrhage: Molecular events and potential treatments. Fluids Barriers CNS.

[37] Pham N., Fazio V., Cucullo L., Teng Q., Biberthaler P., Bazarian J.J., Janigro D. (2010). Extracranial sources of S100B do not affect serum levels. PLoS ONE.

[38] Welch R.D., Ellis M., Lewis L.M., Ayaz S.I., Mika V.H., Millis S., Papa L. (2017). Modeling the Kinetics of Serum Glial Fibrillary Acidic Protein, Ubiquitin Carboxyl-Terminal Hydrolase-L1, and S100B Concentrations in Patients with Traumatic Brain Injury. J. Neurotrauma.

[39] Blais Lécuyer J., Mercier É., Tardif P.A., Archambault P.M., Chauny J.M., Berthelot S., Frenette J., Perry J., Stiell I., Émond M. (2021). S100B protein level for the detection of clinically significant intracranial haemorrhage in patients with mild traumatic brain injury: A subanalysis of a prospective cohort study. Emerg. Med. J. EMJ.

[40] Thelin E.P., Zeiler F.A., Ercole A., Mondello S., Büki A., Bellander B.M., Helmy A., Menon D.K., Nelson D.W. (2017). Serial Sampling of Serum Protein Biomarkers for Monitoring Human Traumatic Brain Injury Dynamics: A Systematic Review. Front. Neurol..

[41] Park D.W., Park S.H., Hwang S.K. (2019). Serial measurement of S100B and NSE in pediatric traumatic brain injury. Child’s Nervous System..

[42] Rogan A., O’Sullivan M.B., Holley A., McQuade D., Larsen P. (2022). Can serum biomarkers be used to rule out significant intracranial pathology in emergency department patients with mild traumatic brain injury? A Systemic Review & Meta-Analysis. Injury.

[43] Rizoli S.B., Jaja B.N., Di Battista A.P., Rhind S.G., Neto A.C., da Costa L., Inaba K., da Luz L.T., Nascimento B., Perez A. (2017). Catecholamines as outcome markers in isolated traumatic brain injury: The COMA-TBI study. Crit. Care.

[44] Feldt-Rasmussen U., Klose M.C. (2025). Pathophysiology and diagnosis of neuroendocrine abnormalities in patients with traumatic brain injury. Best. Pract. Res. Clin. Endocrinol. Metab..

[45] Johnson N.H., Hadad R., Taylor R.R., Rodríguez Pilar J., Salazar O., Llompart-Pou J.A., Dietrich W.D., Keane R.W., Pérez-Bárcena J., Vaccari J.P.d.R. (2022). Inflammatory Biomarkers of Traumatic Brain Injury. Pharmaceuticals.

[46] Ciryam P., Gerzanich V., Simard J.M. (2023). Interleukin-6 in Traumatic Brain Injury: A Janus-Faced Player in Damage and Repair. J. Neurotrauma.

[47] Godeiro Coelho L.M., Teixeira F.J.P., Koru-Sengul T., Manolovitz B., Taylor R.R., Massad N., Kottapally M., Merenda A., Pareja J.C.M., Vaccari J.P.d.R. (2025). Predictive Value of Nervous Cell Injury Biomarkers in Moderate-to-Severe Traumatic Brain Injury: A Network Meta-Analysis. Neurology.

[48] Jalali R., Bałuch M., Malinowska J., Zwiernik J., Kern A., Bil J., Romaszko J. (2025). GFAP/UCH-L1 as a Biomarker for Rapid Assessment of Mild TBI in Emergency Departments. Med. Sci. Monit..

[49] Korley F.K., Jain S., Sun X., Puccio A.M., Yue J.K., Gardner R.C., Wang K.K.W., Okonkwo D.O., Yuh E.L., Mukherjee P. (2022). Prognostic value of day-of-injury plasma GFAP and UCH-L1 concentrations for predicting functional recovery after traumatic brain injury in patients from the US TRACK-TBI cohort: An observational cohort study. Lancet Neurol..

